# Comparative Study of Hemoglobin Values Measured by Continuous Non-invasive and Whole Blood Hemoglobin Monitors for Guiding Blood Transfusion in Major Oncosurgical Procedures

**DOI:** 10.7759/cureus.82616

**Published:** 2025-04-20

**Authors:** Akmal Khan, Shilpi Misra, Virendra Kumar, Shivani Rastogi, Deepak Malviya

**Affiliations:** 1 Anesthesiology, Dr. Ram Manohar Lohia Institute of Medical Sciences, Lucknow, IND

**Keywords:** blood transfusion, hemoglobin, invasive, non-invasive, surgical oncology

## Abstract

Background: Patients undergoing major oncological procedures are at risk of significant perioperative blood loss, leading to increased morbidity and mortality. Transfusions of blood products are associated with complications and adverse effects. Invasive (HemoCue® Hb 301 System (HemoCue AB, Angelholm, Sweden)) and continuous non-invasive (Masimo Radical-7 SpHb® (Masimo Corporation, CA, USA)) monitoring devices have been used as real-time hemoglobin monitors to guide blood transfusion.

Aim: Our study aims to compare the values of both devices for guiding blood transfusion in major abdominal oncosurgical procedures.

Methods: The study was conducted over 18 months on 32 patients scheduled for elective major abdominal oncological procedures. Patients were quasi-randomized for hemoglobin measurement into two groups. Group I (HemoCue® Hb 301 System) (n=16): Invasive values were obtained at baseline and intraoperatively (every 30 minutes). Group II (Masimo Radical-7 SpHb®) (n=16): Continuous non-invasive hemoglobin monitor. Both groups were compared for the measurement of hemoglobin. IBM SPSS Statistics for Windows, Version 21 (Released 2012; IBM Corp., Armonk, New York, United States) was used for statistical analysis.

Results: The mean hemoglobin of the Masimo Radical-7 SpHb® group and the HemoCue® Hb 301 System group during the intraoperative period was not significantly different between the two groups. The blood products transfused between the Masimo Radical-7 SpHb® and the HemoCue® Hb 301 System group were comparable (p=0.917).

Conclusion: Patients of the Masimo Radical-7 SpHb® (non-invasive) had comparable hemoglobin values to the HemoCue® Hb 301 System group (invasive) throughout the surgery. The units of packed red blood cells (PRBC) transfusions were comparable in both groups. The Masimo Radical-7 SpHb® group had the added benefit of being non-invasive with no biohazard exposure.

## Introduction

Risk of bleeding is a significant concern in patients undergoing oncological surgery due to the tumor biology, preoperative cancer modality (chemoradiotherapy and immunotherapy), and perioperative factors such as hemodilution, blood loss, and hypothermia. To rapidly deal with critical hemorrhage, cooperation between anesthesiologists and surgeons and rapid decisions for blood transfusion are required [1]. Evolving surgical oncology procedures, effective anesthesia techniques, optimized perioperative management, and postoperative intensive care modalities have facilitated tumor removal, once considered difficult or not recommended for surgery traditionally [2]. Patients undergoing major oncological surgery are at risk of significant intraoperative and postoperative blood loss, leading to increased morbidity and mortality [3].

Transfusions of blood products are associated with some life-threatening complications and adverse effects, which could be avoided if unnecessary transfusions are not done. Decreased immune surveillance as a result of blood transfusions is also associated with cancer recurrence and progression [4,5]. Therefore, the benefits of red blood cell transfusions during the perioperative period should outweigh the risks associated with the administration of blood products. Maintaining hemoglobin within the target range during surgery by titrated transfusion limits the potential adverse effects of anemia and transfusion-related complications [6,7].

Laboratory hemoglobin has been the primary indicator for blood transfusion. Perioperative transfusion judgments are usually guided by visual estimation of surgical blood loss exceeding maximum allowable blood loss measured by volumetric/gravimetric method, hemodynamic instability despite fluid resuscitation, and periodic hemoglobin measurement using invasive means, such as clinical laboratory testing or arterial blood gas for determination of hemoglobin level.

 A blood sample taken during the early stage of acute blood loss may not indicate the overall hemoglobin level. Therefore, a reliable real-time monitor is needed to guide blood and fluid resuscitation. Invasive measurements are accurate but time-consuming due to the time required to draw and transport the samples to the laboratory, analyze them, and convey the results. HemoCue® Hb 301 System (HemoCue AB, Angelholm, Sweden) is a useful invasive device that is portable, easy to use, and relatively inexpensive for quantitative point-of-care hemoglobin monitoring [8]. It requires only a tiny capillary or venous blood drop and provides an immediate numerical hemoglobin value. Studies have shown that HemoCue® Hb 301 System data is comparable to automated hematology analyzers. However, the HemoCue® Hb 301 System does not provide continuous data and requires blood samples to measure hemoglobin.

Continuous, non-invasive hemoglobin (Masimo Radical-7 SpHb® (Masimo Corporation, CA, USA)) monitoring is now possible with pulse CO-oximetry and multi-wavelength sensors that allow determining hemoglobin levels without sampling or delays [9]. Hemoglobin levels can be monitored continuously during anesthesia, which helps identify potential or occult bleeding and prepares for blood transfusions for improved quality of care. Spectrophotometry hemoglobin (SpHb) monitoring provides continuous, real-time visibility of whether hemoglobin is stable or decreasing; previous studies have shown that hemoglobin values with non-invasive continuous hemoglobin monitors are comparable to lab hemoglobin analyzers. Our study aims to compare the hemoglobin values of a non-invasive continuous hemoglobin monitor, Masimo Radical-7 SpHb®, with an invasive hemoglobin monitor, HemoCue® Hb 301 System, for guiding blood transfusion in major abdominal oncosurgical procedures.

## Materials and methods

After getting approval from the ethical committee (IEC NO. 156/20), the study was conducted at Dr. Ram Manohar Lohia Institute of Medical Sciences on 32 patients posted for elective major abdominal oncosurgical procedures. The study was done over 18 months. Patients aged between 18 and 60 years of either sex with a hemoglobin level >10 gm/dl were included in the study. Patients with any preoperative cardiorespiratory involvement, documented peripheral vascular disease, hemoglobinopathies, and a BMI > 30 were excluded from the study. Informed consent was obtained from all patients. Subjects were quasi-randomized for hemoglobin measurement into two groups: Group I (HemoCue® Hb 301 System) (n=16) and Group II (Masimo Radical-7 SpHb®) (n=16).

After a thorough preoperative anesthetic assessment and required investigations, the patients were posted for elective surgery. On arrival in the operating theater, an electrocardiogram, pulse oximetry, and non-invasive blood pressure were applied; a wide-bore intravenous cannula was secured; general anesthesia was induced in all patients with inj. midazolam (0.07-0.15 mg/kg), inj. fentanyl (1-2 mcg/kg), inj. propofol (1-2.5 mg/kg), and inj. vecuronium (0.08-0.1 mg/kg); an oral cuffed endotracheal tube was inserted using direct laryngoscopy; and the patient was put on mechanical ventilation. Maintenance of anesthesia was done with oxygen:air (40:60), isoflurane (minimum anesthetic concentration (MAC)-1%), and intermittent doses of vecuronium (0.01 mg/kg). After induction and endotracheal intubation, an indwelling urinary catheter was inserted, and a radial arterial catheter was placed for invasive blood pressure monitoring and arterial blood gas analysis. Patients in Group I were monitored for intraoperative hemoglobin value by intermittent blood sampling. An initial blood sample was taken prior to surgery (baseline hemoglobin) in all patients. Intraoperatively, blood samples were taken every 30 minutes or during periods of excessive blood loss. In Group II, patients were monitored with a continuous non-invasive hemoglobin monitor. After induction of anesthesia, the sensor was placed on the ring finger of the non-dominant hand contralateral to the arterial line. Transfusion of blood products was commenced at hemoglobin level <8 gm/dl or hemodynamic instability, i.e., systolic blood pressure (SBP) < 90 mmHg, mean arterial pressure (MAP) < 55 mmHg, or pulse rate > 120 bpm despite fluid resuscitation at the discretion of the attending anesthesiologist. Total blood loss and total blood and fluid (crystalloid and colloid) transfused, along with the patient's heart rate, blood pressure, MAP, plethysmography variability index, and perfusion index, were also recorded. At the end of the surgery, the inhalational anesthetic was discontinued. Neuromuscular blockade was reversed with neostigmine (0.04-0.08 mg/kg) and glycopyrrolate IV (0.005-0.01 mg/kg). After extubation, patients were transferred to the post-anesthetic care unit (PACU) and monitored for hemodynamic parameters.

Statistical analysis

The IBM SPSS Statistics for Windows, Version 21 (Released 2012; IBM Corp., Armonk, New York, United States) was used for statistical analysis. Data was presented as mean (standard deviation) and percentage. The chi-square test was used to compare the dichotomous/categorical variables. More than two groups were compared by ANOVA. A value of P < 0.05 will be considered statistically significant.

## Results

While comparing the demographic data between both groups, the findings were comparable and not statistically significant (p-value=0.329). 

Both groups were comparable on the basis of surgical diagnosis (p=0.260) (Table 1) and surgery performed (p-value=0.548) (Table 2). The mean duration of surgery (hrs) was 5.69 ± 1.00 hrs in Group II and 4.81 ± 0.83 hrs in Group I, and it was statistically significant (p=0.012).

**Table 1:** Comparison of the diagnosis of patients in the Masimo Radical-7 SpHb® group and the HemoCue® Hb 301 System group

Diagnosis	Masimo Radical-7 SpHb® (n=16)		HemoCue® Hb 301 System (n=16)		Chi-sq.	P-value
	n	%	n	%		
Carcinoma ascending colon	2	12.50	2	12.50	11.24	0.260
Carcinoma cervix	0	0.00	2	12.50		
Carcinoma endometrium	1	6.25	0	0.00		
Carcinoma gall bladder	3	18.75	0	0.00		
Carcinoma ovary	1	6.25	2	12.50		
Carcinoma prostate	0	0.00	1	6.25		
Carcinoma stomach	0	0.00	1	6.25		
Carcinoma urinary bladder	2	12.50	5	31.25		
Liver cholangiocarcinoma	2	12.50	1	6.25		
Periampullary carcinoma	5	31.25	2	12.50		

**Table 2:** Comparison of the surgical procedures in the Masimo Radical-7 SpHb® group and the HemoCue® Hb 301 System group

Surgery	Masimo Radical-7 SpHb® (n=16)		HemoCue® Hb 301 System (n=16)		Chi-sq.	P-value
	n	%	n	%		
Interval cytoreduction	2	12.50	2	12.50	6.90	0.548
Left hepatectomy	2	12.50	1	6.25		
Distal gastrectomy	0	0.00	1	6.25		
Lower anterior resection	1	6.25	0	0.00		
Radical cholecystectomy	3	18.75	4	25.00		
Radical cystectomy + ileal conduit (IC)	2	12.50	1	6.25		
Right hemicolectomy	1	6.25	4	25.00		
Whipple procedure	5	31.25	2	12.50		
Radical prostatectomy	0	0.00	1	6.25		

While comparing the mean hemoglobin between both the groups, the maximum hemoglobin in the Masimo Radical-7 SpHb® group was 12.36 + 0.91 at zero min, and the minimum was 7.5 + 0.14 at the end of surgery, while it was a maximum of 12.57 + 1.18 at zero min and the minimum was 7.8 at the end of surgery in the HemoCue® Hb 301 System group. The mean hemoglobin was not significantly different between groups at each time interval except six hours and 30 minutes, at which hemoglobin was slightly higher in the HemoCue® Hb 301 System group (Table 3). 

**Table 3:** Comparison of the mean hemoglobin between the Masimo Radical-7 SpHb® group and the HemoCue® Hb 301 group at different intervals * indicates statistically significant P-value

Hemoglobin	Masimo Radical-7 SpHb® (n=16)			HemoCue® Hb 301 (n=16)			Chi-sq.	P-value
	n	Mean	±SD	n	Mean	±SD		
0 min	16	12.36	0.91	16	12.57	1.18	-0.55	0.583
30 min	16	12.14	0.96	16	12.26	1.24	-0.29	0.775
1 hour	16	11.83	0.92	16	11.91	1.24	-0.23	0.822
1 hr 30 min	16	11.54	1.07	16	11.68	1.20	-0.34	0.735
2 hrs	16	11.26	1.10	16	11.33	1.21	-0.15	0.879
2hrs 30 min	16	10.86	1.34	16	11.01	1.21	-0.33	0.741
3 hrs	16	10.53	1.40	16	10.66	1.24	-0.29	0.771
3hrs 30min	16	10.07	1.47	16	10.28	1.29	-0.43	0.667
4 hrs	16	9.70	1.63	16	9.99	1.40	-0.55	0.589
4hrs 30 min	15	9.35	1.75	11	9.19	1.46	0.25	0.805
5 hrs	15	8.91	1.63	5	8.61	1.33	0.45	0.660
5hrs 30 min	12	8.50	1.58	3	7.80	1.04	0.72	0.485
6 hrs	10	8.09	1.20	2	7.95	0.78	0.16	0.879
6 hrs 30 min	5	7.52	0.11	1	8.30	.	-6.50	0.003^*^
7 hrs (till end of surgery)	5	7.50	0.14	1	7.80	.	-1.94	0.125

The mean maximum allowable blood loss (MABL) (ml) was 603.63 ± 123.21 and 715.31 ±, and the mean blood loss (ml) was 1066.88 ± 459.66 and 1015.00 ± 258.08 in the Masimo Radical-7 SpHb® group and the HemoCue® Hb 301 System group, respectively. The mean MABL (ml) was significantly lower (p=0.015) in the Masimo Radical-7 SpHb® group as compared to the HemoCue® Hb 301 System group, which could be due to the variable body weight of the patient. The blood loss (ml) was higher in the Masimo Radical-7 SpHb® as compared to the HemoCue® Hb 301 System, but was not found to be statistically significant (p-value=0.723 ).

While comparing the amount of blood products transfused between the Masimo Radical-7 SpHb® group and the HemoCue® Hb 301 System group, both groups were comparable, and findings were not found to be statistically significant (p-value=0.917 for packed red blood cells) (Table 4).

**Table 4:** Comparison of blood products transfused between the Masimo Radical-7 SpHb® group and the HemoCue® Hb 301 System group PRBC: packed red blood cells; FFP: fresh frozen plasma

Blood Products (Unit)		Masimo Radical-7 SpHb® (n=16)		HemoCue® Hb 301 (n=16)		Chi-sq.	P-value
		n	%	n	%		
PRBC	Nil	8	50.00	6	37.50	0.95	0.917
	1 unit	1	6.25	1	6.25		
	2 unit	2	12.50	4	25.00		
	3 unit	4	25.00	4	25.00		
	4 unit	1	6.25	1	6.25		
FFP	Nil	6	37.50	8	50.00	1.68	0.431
	2 unit	4	25.00	3	18.75		
	4 unit	6	37.50	5	31.25		

## Discussion

Massive blood loss during major oncological surgeries is a concern for both surgeons and anesthesiologists. Accurate estimation of blood loss is essential because underestimation can lead to significant complications, and overestimation and unnecessary blood transfusions can increase morbidity and mortality [10]. Balancing the need for red blood cell transfusions to alleviate anemia with the risks associated with administering blood products can be a complex aspect of perioperative care. There are many ways to estimate intraoperative blood loss, e.g., gravimetry (the gauze is weighed before and after use), photometry (via photometry of blood concentrations in hemolyzed irrigating fluid), and visual estimation, but the assessment is least accurate.

Perioperative real-time measurement of hemoglobin can assess the amount of blood loss and thus guide blood product transfusion. In addition to laboratory hemoglobin levels, point-of-care devices such as the HemoCue® Hb 301 System can be used in the operating room to measure hemoglobin levels. However, this is invasive and requires intermittent blood sample analysis.

While the automated lab hemoglobin analyzer has the disadvantage of invasive blood sampling and time delay in transportation and reporting, HemoCue® Hb 301 System (invasive but on-the-spot hemoglobin values) and continuous non-invasive hemoglobin monitoring (Masimo Radical-7 SpHb®) provide clinicians with real-time updated information on hemoglobin trends. It can change the decision for red blood cell transfusions [11,12]. This study aimed to evaluate the utility of a non-invasive hemoglobinometer (Masimo Radical-7 SpHb®) and an invasive hemoglobinometer (HemoCue® Hb 301 System) in guiding perioperative blood transfusion during oncosurgery, thus preventing unnecessary blood transfusion.

Demographic values, including age and sex, were comparable in both groups and were not found to be statistically significant. The surgical procedures, including major open abdominal oncolosurgical procedures, were similar in both groups (p-value=0.548).

The mean duration of surgery (hrs) was significantly longer in the Masimo Radical-7 SpHb® group than in the HemoCue® Hb 301 System group (p=0.012). The mean hemoglobin was not significantly different between groups at each time interval except at 30 min, at which hemoglobin was slightly higher in the HemoCue® Hb 301 System group than in the Masimo Radical-7 SpHb® group (p=0.003). Our study also observed that the drop in hemoglobin was significant from zero minutes till the end of surgery in the Masimo Radical-7 SpHb® group (p < 0.001). The maximum hemoglobin in the Masimo Radical-7 SpHb® group was 12.36 + 0.91 at zero minutes, and the minimum was 7.5 + 0.14 at the end of surgery.

The mean change in the pleth variability index (PVi) in Masimo Radical-7 SpHb® significantly increased from zero min till the end of surgery (p < 0.001). Still, the change lay under the average value for the PVi, which is <13%, suggesting fluid responsiveness.

Miller RD et al. (2011) made a comparison of three methods (SpHb, HemoCue® Hb 301 System Hb, and Lab Hb) of hemoglobin monitoring among 20 patients who underwent spine surgery [13]. They concluded that the HemoCue® Hb 301 System was consistently accurate and SpHb often correlated well with lab hemoglobin values. Shah et al. compared the accuracy of non-invasive and invasive point-of-care total blood hemoglobin measurement in an outpatient setting [14]. They also found comparable results in both groups and concluded that SpHb measurement, being non-invasive, may offer additional benefits for both patients and providers. Our study also found the mean hemoglobin values comparable between both groups. Lamhaut et al. (2011) did a comparison of the accuracy of non-invasive hemoglobin monitoring by SpHb and HemoCue® Hb 301 System with automated laboratory hemoglobin measurement among 44 patients [15]. They found that the precision of hemoglobin measured by the HemoCue® Hb 301 System was significantly better than that of continuous hemoglobin (SpHb). Our study found the readings by invasive (HemoCue® Hb 301 System) and non-invasive (SpHb) comparable at all time intervals. Both groups were similar when compared for the blood products used between the Masimo Radical-7 SpHb® group and the HemoCue® Hb 301 System group (p=0.917). 

Saracoglu et al. (2022) measured hemoglobin continuously during frontal advancement operations [16]. They also found that patients with perioperative continuous SpHb measurement had lower intraoperative PRBC transfusion and concluded that SpHb, together with clinical judgment, can be used in decision-making for perioperative PRBC transfusion, which correlates with the results of our study.

Awada W.N. et al. (2015) conducted a prospective cohort study regarding continuous and non-invasive hemoglobin monitoring and concluded that adding SpHb monitoring to the standard-of-care blood management decreased blood utilization in high blood loss neurosurgery while facilitating earlier transfusions [17]. We also used the reading of continuous hemoglobin monitoring as a guide for blood transfusion preoperatively. Various other authors have used non-invasive hemoglobin monitoring as a guide to the decision of blood transfusion in major surgeries and found it a suitable alternative to intermittent invasive testing [18,19].

In our study, we found that patients of the Masimo Radical-7 SpHb® group (non-invasive continuous hemoglobinometer) had comparable hemoglobin readings to the HemoCue® Hb 301 System group (invasive hemoglobinometer). The units of PRBC transfusions were comparable in both groups. The Masimo Radical-7 SpHb® group had the added benefit of being non-invasive, with no biohazard exposure and no requirement for a curette for hemoglobin measurement, as with the HemoCue® Hb 301 System. The Masimo Radical-7 SpHb® group was also monitored for changes in the perfusion index (Pi) and PVi throughout the surgery, which helped guide fluid responsiveness.

Limitations

Our study included a relatively smaller sample size; further studies with bigger sample sizes could authenticate the findings more. The Masimo Radical-7 SpHb® device and HemoCue® Hb 301 System were not independently compared with the laboratory automated hemoglobin analyzer, which is the standard measurement device for hemoglobin in hospital settings.

## Conclusions

In our study, we found that both groups had comparable hemoglobin readings and units of PRBC transfusions. The Masimo Radical-7 SpHb®group had the added benefit of being non-invasive with no biohazard exposure and no requirement for a curette for hemoglobin measurement. The Masimo Radical-7 SpHb® group was also monitored for changes in Pi and PVi throughout the surgery, which helped in guiding fluid responsiveness during the surgery.

We conclude that real-time hemoglobin monitoring by a continuous hemoglobin monitor (Masimo Radical-7 SpHb®), along with clinical assessment, can be used to guide decisions regarding blood transfusions in major surgical procedures.
